# Overexpression of *Arabidopsis P3B* increases heat and low temperature stress tolerance in transgenic sweetpotato

**DOI:** 10.1186/s12870-017-1087-2

**Published:** 2017-08-14

**Authors:** Chang Yoon Ji, Rong Jin, Zhen Xu, Ho Soo Kim, Chan-Ju Lee, Le Kang, So-Eun Kim, Hyeong-Un Lee, Joon Seol Lee, Chang Ho Kang, Yong Hun Chi, Sang Yeol Lee, Yiping Xie, Hongmin Li, Daifu Ma, Sang-Soo Kwak

**Affiliations:** 1 Plant Systems Engineering Research Center, Korea Research Institute of Bioscience and Biotechnology (KRIBB), 125 Gwahak-ro, Daejeon, 34141 South Korea; 2 Department of Environmental Biotechnology, Korea University of Science and Technology (UST), 217 Gajeong-ro, Daejeon, 34113 South Korea; 3 Sweetpotato Research Center, Jiangsu Academy of Agricultural Science, Xuhuai Road, Xuzhou, Jiangsu 221131 China; 4 Bioenergy Crop Research Center, National Institute of Crop Science, Rural Development Administration, Muan, 58545 South Korea; 5 Division of Applied Life Science (BK21 Plus program) and Plant Molecular Biology and Biotechnology Research Center, Gyeongsang National University, 501 Jinjudae-ro, Jinju, 52828 South Korea

**Keywords:** Acidic ribosomal P-proteins, Heat stress, Low temperature stress, Protein chaperone, Sweetpotato

## Abstract

**Background:**

Sweetpotato (*Ipomoea batatas* [L.] Lam) is suitable for growth on marginal lands due to its abiotic stress tolerance. However, severe environmental conditions including low temperature pose a serious threat to the productivity and expanded cultivation of this crop. In this study, we aimed to develop sweetpotato plants with enhanced tolerance to temperature stress.

**Results:**

P3 proteins are plant-specific ribosomal P-proteins that act as both protein and RNA chaperones to increase heat and cold stress tolerance in *Arabidopsis*. Here, we generated transgenic sweetpotato plants expressing the *Arabidopsis* ribosomal P3 (*AtP3B*) gene under the control of the CaMV 35S promoter (referred to as OP plants). Three OP lines (OP1, OP30, and OP32) were selected based on *AtP3B* transcript levels. The OP plants displayed greater heat tolerance and higher photosynthesis efficiency than wild type (WT) plants. The OP plants also exhibited enhanced low temperature tolerance, with higher photosynthesis efficiency and less membrane permeability than WT plants. In addition, OP plants had lower levels of hydrogen peroxide and higher activities of antioxidant enzymes such as peroxidase and catalase than WT plants under low temperature stress. The yields of tuberous roots and aerial parts of plants did not significantly differ between OP and WT plants under field cultivation. However, the tuberous roots of OP transgenic sweetpotato showed improved storage ability under low temperature conditions.

**Conclusions:**

The OP plants developed in this study exhibited increased tolerance to temperature stress and enhanced storage ability under low temperature compared to WT plants, suggesting that they could be used to enhance sustainable agriculture on marginal lands.

**Electronic supplementary material:**

The online version of this article (doi:10.1186/s12870-017-1087-2) contains supplementary material, which is available to authorized users.

## Background

To cope with climate change and environmental stresses such as drought, temperature variation, UV radiation, and salinity, plants have evolved sophisticated signaling and protective systems [1, 2]. Since plants are sessile organisms, they must utilize various mechanisms to respond and adapt to continuously changing environmental conditions [3–5]. Temperature variation is an especially important environmental factor that affects plant development and crop production [6, 7]. Understanding the molecular mechanisms underlying the plant response to various stresses has been a subject of great interest for many decades. Nevertheless, there is still a significant knowledge gap and, in general, we are unable to predict how well plants will cope with multiple environmental stress factors.

Sweetpotato (*Ipomoea batatas* [L.] Lam) is an important root crop worldwide [8, 9]. This crop is used as an alternative source of animal feed and industrial biomass for biomaterial and biofuel production, and it represents an abundant source of nutrients and natural antioxidant compounds for the human diet, such as anthocyanins, carotenoids, and Vitamin C and E [10–13]. Sweetpotato is well suited for growth on marginal lands due to its tolerance to abiotic stress [14]. Thus, sweetpotato represents an industrially valuable source of biomass to supplement grain-based bioenergy production and increase food security. Nevertheless, sweetpotato as a tropical crop is very sensitive to low temperature, posing critical threats to the productivity and geographical distribution. Opportunities for genetic improvement of sweetpotato using conventional breeding are limited due to its high male sterility, hexaploid nature, the lack of suitable germplasm, and issues with incompatibility [15, 16]. Thus, it is highly important to develop a sweetpotato cultivar with enhanced tolerance to severe abiotic stresses via genetic engineering.

To overcome temperature stress, plants must immediately recognize the outside temperature and communicate this information via signaling cascades, which activate distinct downstream proteins such as heat-shock proteins (HSPs) and cold-shock proteins (CSPs), initiating downstream temperature stress-related responses [17]. In sweetpotato, a number of genes have been identified that are involved in the responses to abiotic stresses, such as drought, oxidative, and salt stress [18–25]. However, little is known about the temperature stress response in sweetpotato.

A distinct lateral protuberance of the large ribosomal subunit in eukaryotic ribosomes called the “stalk”, which contains highly conserved small ribosomal proteins with an acidic isoelectric point (pI 3–5) [26, 27]. Acidic ribosomal proteins (ARPs) are phosphorylated by several protein kinases to facilitate assembly into ribosomes [28] and, therefore, these proteins are also referred to as ribosomal P-proteins. P-proteins in various eukaryotes have three domains, including an α-helical N-terminal region and a central, flexible acidic hinge region, followed by a highly conserved C-terminus [27]. In all eukaryotes, these proteins are categorized into two groups, P1 and P2, based on primary sequence similarity [29], with the exception of plants, which contain an extra group, P3 [30]. The “stalk” is the active part of the ribosome structure and the center for interactions between mRNAs, tRNAs, and translation factors occur during protein synthesis [31]. The biological functions of eukaryotic ribosomal P-proteins are currently unclear. P-proteins are believed to function in the regulation of protein synthesis at the level of the protein elongation step. These proteins might also be involved in transcriptional processes and DNA repair [32]. In addition, phosphorylation is an important posttranslational step that regulates P-protein function [27]. Conserved phosphorylation sites have been identified in *Arabidopsis* [33] and maize [34, 35]. However, the biological significance of plant P-proteins has not been fully elucidated.

Recently, Kang et al. identified a plant-specific acidic ribosomal P3 protein (designated AtP3B) as a heat-shock protein from heat-treated *Arabidopsis* suspension cells [36]. The authors demonstrated that AtP3B has both protein and RNA chaperone activities. Overexpressing *AtP3B* increased tolerance to high- and low temperature stress in transgenic plants, whereas knockdown plants of *AtP3B* created by RNAi showed increased sensitivity to both stresses. Here, we developed transgenic sweetpotato plants that overexpressed *AtP3B* and evaluated their growth under heat and low temperature stress. Transgenic sweetpotato overexpressing *AtP3B* showed not only increased temperature stress tolerance, but also improved storage ability under low temperature stress conditions.

## Results

### Molecular characterization of transgenic sweetpotato overexpressing *AtP3B*

**Fig. 1:**
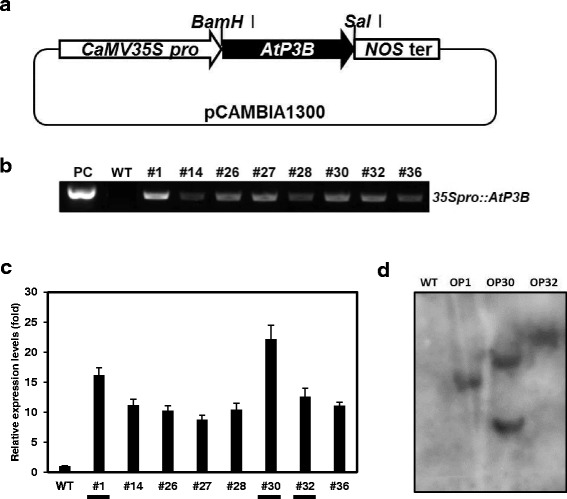
Development and molecular characterization of transgenic sweetpotato plants overexpressing *AtP3B*. **a** Schematic diagram of the vector construct containing *AtP3B* under the control of the CaMV 35S promoter. **b** Genomic DNA PCR analysis using the *35Spro::AtP3B* primer set. PC, positive control. **c** qRT-PCR analysis of transgenic sweetpotato plants overexpressing *AtP3B*. Three independent transgenic lines (#1, #30, and #32) were selected for further characterization. **d** Southern blot analysis of OP plants; the integration and gene copy number of the construct in OP plants were confirmed using a ^32^P–labeled probe designed based on the AtP3B cDNA fragment

Transgenic sweetpotato plants overexpressing *AtP3B* under the control of the CaMV 35S promoter (Fig. 1a) were successfully generated via *Agrobacterium*-mediated transformation. We performed an initial screening of the putative transgenic sweetpotato plants using PCR analysis of genomic DNA with a portion of the 35S promoter and *AtP3B*-specific primers. The expected amplification profiles were acquired from eight transgenic lines, suggesting that the recombinant *AtP3B* gene had been integrated into the genomes of transgenic plants from eight independent lines, whereas no integration was detected in the wild type (WT) line (Fig. 1b). Transgenic plants harboring *AtP3B* under the control of the CaMV 35S promoter were designated “OP” plants. We propagated the eight independent OP lines in a growth chamber and subjected the plants to quantitative RT-PCR analysis using leaf discs to determine the transcription levels of *AtP3B* (Fig. 1c). *AtP3B* expression was strongly induced in lines OP1, OP30, and OP32 (Fig. 1c); we therefore selected these lines for further study. As shown in Fig. 1d, lines OP1, OP30, and OP32 contained single, double, and single copy insertions of AtP3B, respectively. When OP transgenic and WT plants were grown in the growth chamber, no visible phenotypic differences were detected under normal conditions (data not shown), indicating that *AtP3B* overexpression did not lead to phenotypic defects in transgenic sweetpotato plants.

### Transgenic sweetpotato overexpressing *AtP3B* display enhanced tolerance to heat and low temperature stress

**Fig. 2:**
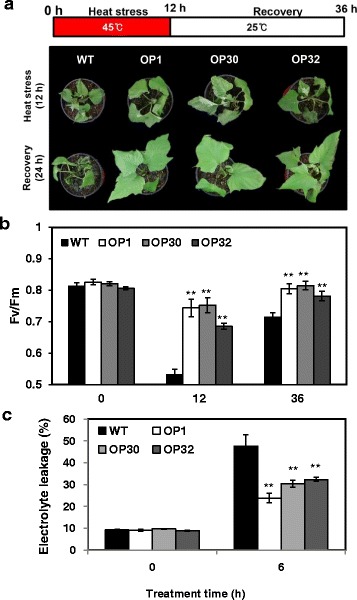
Phenotypic and physiological analyses of OP plants under heat stress treatment (45 °C) and after recovery at 25 °C. **a** Visible damage in the leaves of sweetpotato plants after 12 h heat stress treatment and 24 h recovery. **b** PSII photosynthetic efficiency (*Fv/fm*) in the leaves of WT and OP plants after 12 h heat stress treatment and 24 h recovery. **c** Analysis of electrolyte leakage. Data are expressed as the mean ± SD of three replicates. *Asterisks* indicate significant differences between WT and OP plants by ANOVA at * *p* < 0.05 and ** *p* < 0.01

*AtP3B*-overexpressing transgenic *Arabidopsis* plants showed enhanced tolerance to heat and low temperature stress, whereas knockdown of *AtP3B* by RNAi led to increased sensitivity to both stresses [36]. In the current study, we evaluated the physiological functions of AtP3B using transgenic sweetpotato plants. First, we investigated the tolerance of WT and OP plants to heat stress conditions. Under normal conditions (25 °C), the phenotypes of OP plants did not differ from those of WT plants in terms of plant growth. However, when we treated 1-month-old WT or OP plants with heat stress (45 °C) for 12 h, the OP plants exhibited marked thermotolerance compared to WT plants (Fig. 2a). Following recovery via incubation at 25 °C, severe damage was observed in WT plants, whereas the OP plants exhibited only slight wilting (Fig. 2a). Under this heat stress condition, the photosynthetic efficiency (*Fv/fm*) of WT plants decreased by 35.3%, whereas the photosynthetic efficiency only decreased by 9.7%, 8.5%, and 15.0% in OP1, OP30, and OP32 plants, respectively (Fig. 2b). After a 24 h recovery, the photosynthetic efficiency of OP plants was maintained to approximately similar levels of normal conditions, whereas WT plants continued to show reduced photosynthetic efficiency. In addition, OP plants exhibited significantly lower levels of ion leakage than WT plants after heat stress treatment (Fig. 2c). These results suggest that OP sweetpotato plants are more tolerant to high temperature stress than WT plants due to overexpression of *AtP3B* in the transgenic plants.

**Fig. 3:**
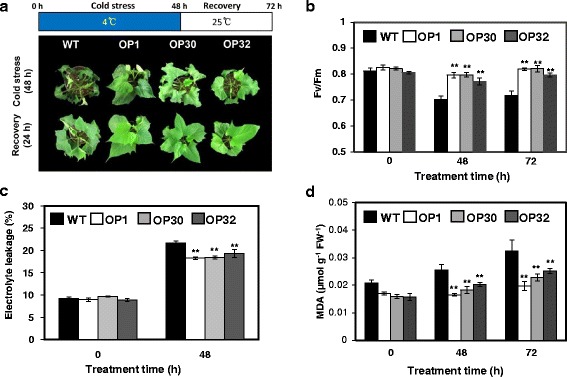
Phenotypic and physiological analyses of OP plants under low temperature treatment (4 °C) and after recovery at 25 °C. **a** Visible damage in the leaves of sweetpotato plants after 48 h cold stress treatment and 24 h recovery. **b** PSII photosynthetic efficiency (*Fv/fm*), **c** Ion leakage in detached leaves treated with 4 °C for 48 h, and **d** MDA contents in the leaves of WT and OP plants after 48 h cold stress treatment and 24 h recovery. Data are expressed as the mean ± SD of three replicates. *Asterisks* indicate significant differences between WT and OP plants by ANOVA at * *p* < 0.05 and ** *p* < 0.01

AtP3B plays an essential role in cold stress tolerance in *Arabidopsis*, a process mediated by its RNA chaperone activity [36]. Therefore, we investigated whether the OP plants also exhibited increased tolerance to low temperatures due to *AtP3B* overexpression. We subjected soil-grown whole plants (1 month old) to low temperature conditions (4 °C) for 48 h, followed by recovery at 25 °C. After low temperature treatment, severe wilting and chilling injury were observed in the leaves of WT plants, whereas all OP plant lines (OP1, OP30, and OP32) showed only slight damage (Fig. 3a). Following a 24 h recovery period, the phenotypes of OP plants had returned to normal. However, WT plants still had slight dehydration symptoms (Fig. 3a). The photosynthetic efficiency (*Fv/fm*) of both WT and OP plants decreased during the low temperature treatment. After exposure to 4 °C conditions for 48 h, the *Fv/fm* values of WT plants decreased by 13.5%, which were significantly lower than those of the three OP lines. The photosynthetic efficiency of all OP plants was restored to normal levels after a 24 h recovery at 25 °C, whereas the WT plants continued to show reduced *Fv/fm* values (Fig. 3b). In addition, OP plants exhibited significantly lower levels of ion leakage than WT plants after low temperature treatment (Fig. 3c). Malondialdehyde (MDA), a naturally occurring product of lipid peroxidation due to accelerated reactive oxygen species (ROS) production, is an important indicator of cell membrane damage under stress conditions [37]. Under normal conditions, the MDA contents of WT plants were similar to those of OP plants. However, after 4 °C treatment for 48 h, MDA levels were higher in WT plants than in OP plants. Following incubation at 25 °C for 24 h, the MDA contents were significantly higher in WT plants than in OP plants (Fig. 3d). These results indicate that the degree of cell membrane damage was greater in WT plants than in OP plants under low temperature stress.

We also examined the expression of *IbHSP*, *IbCBF* and *IbCOR* genes (*IbHSP17.6*, *IbHSP18.2, IbCBF3* and *IbCOR27*), which are candidate homologs of *Arabidopsis HSP, CBF* and *COR* genes. The transgenic sweetpotato plants exhibited higher expression levels of these target genes than did the WT plants (Additional file 1: Fig. S1). The altered expression levels of these *IbHSP*, *IbCBF* and *IbCOR* candidate genes might help explain the enhanced heat and cold stress tolerance of the transgenic sweetpotato plants.

### *AtP3B*-overexpressing transgenic sweetpotato plants show enhanced antioxidant enzyme activity

**Fig. 4:**
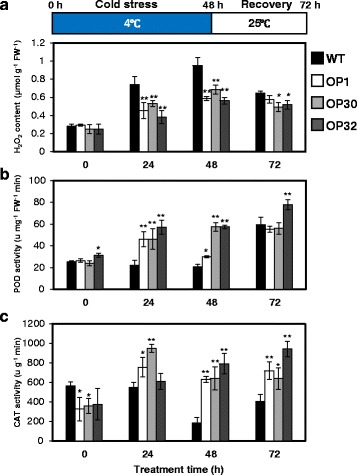
Analysis of H_2_O_2_ contents and antioxidant enzyme activity in WT and OP plants under low temperature stress. **a** H_2_O_2_ contents in leaves of WT and OP plants after 48 h low temperature stress treatment and 24 h recovery. **b** and **c** Changes in antioxidant enzyme activity in WT and OP plants after 48 h low temperature stress treatment and 24 h recovery. POD activity (**b**) and CAT activity (**c**). Data are shown as mean ± SD of three independent measurements. *Asterisks* indicate significant differences between WT and OP plants by ANOVA at * *p* < 0.05 and ** *p* < 0.01

Low temperature stress induces H_2_O_2_ accumulation, which can severely damage cells [38]. Thus, we investigated the H_2_O_2_ contents in WT versus OP plants after low temperature treatment. Under normal growth conditions, the H_2_O_2_ contents in OP and WT plants were similar (Fig. 4a). After low temperature treatment at 4 °C for 24 h and 48 h, the H_2_O_2_ levels in WT plants were 1.6- to 1.5-fold higher than those of OP (OP1, OP30, and OP32) plants (Fig. 4a). After 24 h recovery, the H_2_O_2_ contents in WT plants were still significantly higher than those of OP plants (Fig. 4a). These results indicate that *AtP3B* expression suppresses H_2_O_2_ accumulation under low temperature stress in transgenic sweetpotato plants.

Exposure of plants to unfavorable environmental conditions leads to the overproduction of ROS, which can cause significant oxidative damage to proteins, lipids, carbohydrates, and DNA [39]. ROS-scavenging enzymes such as superoxide dismutase (SOD), ascorbate peroxidase (APX), catalase (CAT), and peroxidase (POD) are very important for plants, as they help protect plants from toxic oxygen intermediates [6, 40, 41]. In particular, POD and CAT are major enzymes responsible for H_2_O_2_ scavenging during oxidative stress in plants. Under normal conditions, the POD activity levels in the three OP lines were similar to that of WT plants (Fig. 4b). However, the POD activity of OP plants significantly increased under low temperature treatment. After exposure to 4 °C for 24 h and 48 h, the OP plants exhibited an average of 2.2- and 2.3-fold higher POD activity than WT plants, respectively (Fig. 4b). Following a 24 h recovery, POD activity levels were similar among WT and OP plants, except for OP32. In addition, under normal conditions, CAT activity was lower in OP plants than in WT plants (Fig. 4c). CAT activity in WT plants was significantly reduced by low temperature treatment, whereas CAT activity in OP plants was not affected by cold stress. After 4 °C treatment for 48 h, the average CAT activity level was 3.7-fold higher in OP plants than in WT plants (Fig. 4c). After 24 h of recovery, the OP plants still exhibited higher CAT activity than WT plants (Fig. 4c). These results suggest that the enhanced low temperature stress tolerance of OP plants might be attributed to the increased activity of ROS-scavenging enzymes such as POD and CAT.

### Yield of *AtP3B* transgenic sweetpotato plants under field conditions

**Fig. 5:**
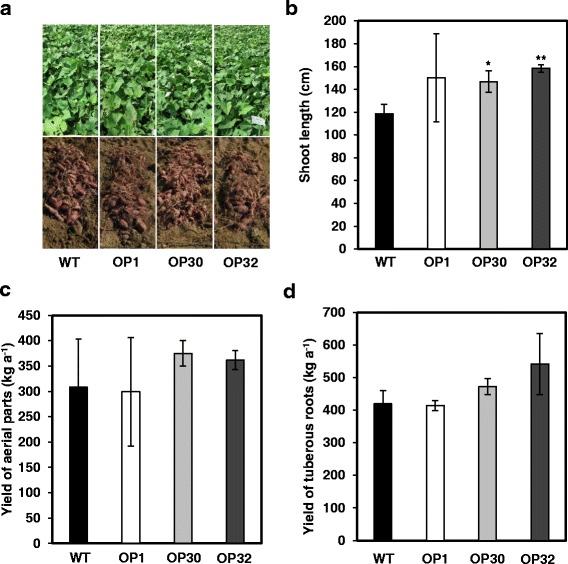
Growth-related features of WT and OP plants under field conditions. **a** Photographs of aerial plant parts and tuberous roots. **b** Average shoot lengths of plants. **c** Average yields of aerial plant parts. **d** Average yields of tuberous roots. Data are means ± SD of three row replicates (40 individual plants were planted per line). *Asterisks* indicate significant differences between WT and OP plants by ANOVA at * *p* < 0.05 and ** *p* < 0.01

To assess whether overexpressing *AtP3B* affects sweetpotato yields, we measured the yields of the aerial parts and tuberous roots of the transgenic lines. Under field conditions, the yields of aerial parts and tuberous roots were not significantly different between WT and OP plants, although one transgenic line (OP32) had slightly higher yields than WT plants (Fig. 5). The average shoot length of WT plants was approximately 118.6 cm, whereas that of OP plants was slightly higher (150.3 cm for OP1, 146.8 cm for OP30, and 158.4 cm for OP32) (Fig. 5b). The average yield for the aerial parts of WT plants was 308 kg per are (a), while OP1, OP30, and OP32 plants produced 299.1, 375, and 361.6 kg a^−1^, respectively (Fig. 5c). Moreover, we evaluated the average yields of tuberous roots, which varied among transgenic lines. The yields of tuberous roots in WT, OP1, OP30, and OP32 plants were similar (419.1 kg a^−1^ for NT, 413.8 kg a^−1^ for OP1, and 472.3 kg a^−1^ for OP30, and 541.5 kg a^−1^ for OP32; Fig. 5d).

### The tuberous roots of OP transgenic sweetpotato show enhanced storage ability under low temperature conditions

**Fig. 6:**
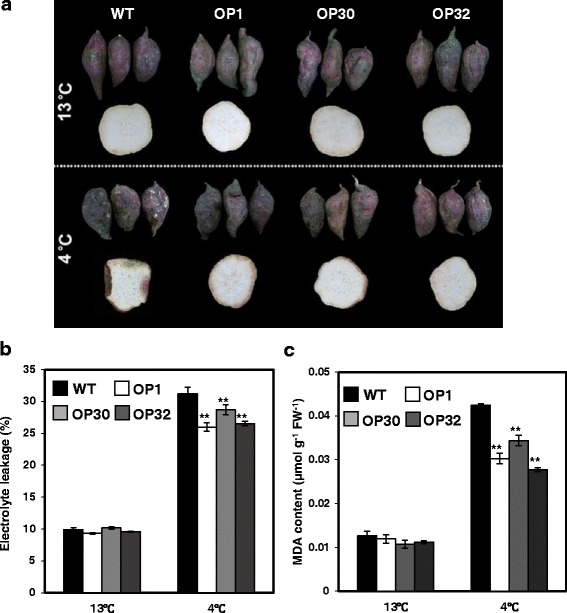
Storage ability of transgenic sweetpotato tuberous roots during low temperature storage. **a** Photographs of WT and OP tuberous roots stored at 13 °C and 4 °C for 8 weeks. **b** Analysis of ion leakage and **c** MDA contents in tuberous roots of WT and OP plants stored in 13 °C and 4 °C for 8 weeks. Data are expressed as the mean ± SD of three replicates. *Asterisks* indicate significant differences between WT and OP plants by ANOVA at * *p* < 0.05 and ** *p* < 0.01

Long-term exposure to low temperature causes a variety of chilling injuries in sweetpotato tuberous roots [42–46]. As mentioned, the OP transgenic lines exhibited increased tolerance to low temperature stress (Fig. 3). To further verify the low temperature stress resistance of OP transgenic sweetpotato, we investigated the physiological responses of tuberous roots from field-grown OP plants under low temperature storage. Sweetpotato tuberous roots from both WT and OP plants showed no symptoms of chilling injury or morphological changes when stored at 13 °C for 8 weeks (Fig. 6a). However, after storage for 8 weeks at 4 °C, severe morphological changes including surface wounding, darkening of internal tissues and susceptibility to decay were observed in tuberous roots of WT plants, whereas all three OP 77 lines (OP1, OP30, and OP32) showed only slight damage (Fig. 6a). In addition, we compared ion leakage levels between WT and OP tuberous roots under long-term exposure to low temperature. The tuberous roots of WT and OP plants exhibited similar levels of ion leakage when stored at 13 °C for 8 weeks (Fig. 6b). However, after incubation at 4 °C for 8 weeks, tuberous roots of OP plants had lower levels of ion leakage than those of WT plants (Fig. 6b). Chilling injuries caused by long-term exposure to low temperature include cellular membrane degradation [42]. Thus, we also measured the MDA contents of tuberous roots, which represent the degree of cell membrane damage, under chilling stress. Under storage at 13 °C for 8 weeks, the MDA contents of OP tuberous roots were similar to those of WT plants. After storage for 8 weeks at 4 °C, the MDA contents of WT tuberous roots were higher than those of OP plants overexpressing *AtP3B* (Fig. 6c). These results indicate that overexpressing *AtP3B* in sweetpotato increases storage ability under low temperature conditions.

## Discussion

*AtP3B*, which was originally isolated from heat-treated *Arabidopsis* suspension culture cells, plays essential roles in both heat and cold tolerance. AtP3B plays dual roles as a protein chaperone and an RNA chaperone; it prevents protein aggregation under heat stress, whereas it supports RNA processing or stability under cold stress [36]. In this study, ectopic expression of *AtP3B* in sweetpotato resulted in enhanced tolerance to heat and low temperature stress. Interestingly, overexpressing *AtP3B* increased the storage ability of this root crop during postharvest storage at low temperature.

Temperature stress, including heat, cold, and freezing stress, poses a major threat to crop productivity [47]. ROS produced by these stresses are toxic molecules capable of causing oxidative damage to proteins, cell membranes, nucleic acids, carbohydrates, and lipids [48]. Under normal growth conditions, ROS are mainly generated at low levels in organelles such as chloroplasts, mitochondria, and peroxisomes, whereas their rate of production dramatically increases during temperature stress. The ROS, H_2_O_2_, plays dual roles in plants: at low concentrations, it acts as a signaling molecule involved in triggering defense responses to various biotic and abiotic stresses, whereas at high concentrations, it induces programmed cell death [49]. In the current study, while H_2_O_2_ levels significantly increased in WT sweetpotato under low temperature stress (Fig. 4a), these levels were lower in OP plants than in WT plants under these conditions. In addition, overexpressing *AtP3B* in sweetpotato increased POD and CAT activity under low temperature stress conditions (Fig. 4b and c). POD and CAT are the major enzymes responsible for H_2_O_2_ scavenging during oxidative stress in plants. In addition to H_2_O_2_ scavenging, plant PODs are also involved in plant growth and development, as well as the lignification, suberization, and cross-linking of cell wall compounds [50]. In addition, CAT eliminates H_2_O_2_ by breaking it down directly to form water and oxygen. Thus, CAT activity does not require reducing power and has a high reaction rate, but it has a low affinity for H_2_O_2_, thereby only removing high concentrations of H_2_O_2_ [51]. Interestingly, CAT activity was significantly higher in OP plants than in WT plants (Fig. 4c). These data are consistent with the notion that the increase in POD and CAT activity resulting from *AtP3B* expression in transgenic sweetpotato plants is correlated with low temperature stress tolerance via an H_2_O_2_-regulated stress response-signaling pathway.

Sweetpotato is a high-yielding, industrially valuable root crop. While sweetpotato is widely adapted to growth on marginal lands ranging from tropical to temperate zones, it is highly sensitive to low temperature stress. In addition, postharvest storage conditions for sweetpotato are a major issue affecting its use for industrial applications sweetpotato. Therefore, it is essential to genetically engineer sweetpotato with enhanced tolerance to low temperatures. We previously produced sweetpotato plants with enhanced tolerance to low temperature stress via genetic engineering. Transgenic sweetpotato plants overexpressing the soybean cold-inducible zinc finger protein gene *SCOF-1* under the control of an oxidative stress-inducible peroxidase (*SWPA2*) promoter exhibited enhanced tolerance to low temperature stress [52]. We also reported that transgenic sweetpotato plants expressing the *Arabidopsis* nucleoside diphosphate kinase 2 gene (*AtNDPK2*) exhibited enhanced tolerance to multiple environmental stresses, including cold, high salt, drought, and MV-mediated oxidative stress, due to increased H_2_O_2_-scavenging enzyme activity regulated by NDPK2 [53]. However, enhanced tolerance to low temperature stress in sweetpotato has not previously been correlated with increased postharvest storage ability under low temperatures. In the current study, we demonstrated that expressing *AtP3B* led to increased H_2_O_2_ scavenging enzyme activity and enhanced tolerance to low and high temperature stress in transgenic sweetpotato. Furthermore, overexpressing *AtP3B* in sweetpotato increased its storage ability at low temperatures. We are currently focused on isolating and characterizing endogenous homologous genes of *AtP3B* in sweetpotato. Such work should pave the way for improving postharvest storage ability in sweetpotato without the loss of quality due to chilling injury.

## Conclusion

In this study, we successfully developed transgenic sweetpotato plants overexpressing *AtP3B* under the control of the CaMV 35S promoter. As expected, the transgenic sweetpotato plants exhibited enhanced tolerance to heat and low temperature stress. After exposure to low temperature stress, the OP plants displayed less wilting and chilling symptoms than WT plants, which was associated with enhanced antioxidant enzyme activity. In addition, the OP plants exhibited a stronger ability to recover from low temperature stress than WT plants. Finally, the tuberous roots of OP transgenic sweetpotato showed enhanced storage ability at low temperatures compared to WT plants.

## Methods

### Plant materials

Sweetpotato (*Ipomoea batatas* [L.] Lam., cv. Xushu 29, one of the most widely grown varieties in Northwest China) plants were used in this study. The plants were cultivated in a growth chamber in soil at 25 °C under a 16 h/8 h (light/dark) photocycle. Embryogenic calli cultured on MS medium containing 1 mg l^−1^ 2,4-dichlorophenoxyacetic acid (2,4-D). The embryogenic calli were proliferated by subculture in fresh medium at 3 week intervals in the dark in a 25 °C incubator.

### Vector construction and transformation

The *AtP3B* gene construct was generated in plant expression vector pCAMBIA1300 using CaMV 35S promoter and a NOS terminator sequence. CaMV 35Spro::AtP3B plasmids were transformed into embryogenic calli from sweetpotato via *Agrobacterium*-mediated transformation as described by Lim et al. [54]. The transformed embryogenic calli were selected on MS medium containing 400 mg l^−1^ cefotaxime, and 25 mg l^−1^ hygromycin, and subcultured in fresh medium at 3 week intervals. After transgenic sweetpotato plants were generated, vine cuttings from the transgenic sweetpotato and WT plants were used for propagation. Regenerated plants were transplanted into pots and grown in a greenhouse for further analysis.

### Southern blot analysis

Up to 30 μg genomic DNA of indicated sweetpotato plants was digested with *Eco*RI (Roche, Manheim, Germany) and separated on a 0.8% agarose gel. The separated DNA was hybridized with an [α-^32^P]dCTP labeled probe after transferred to a positively charged nylon membrane (Bio-Rad, CA, USA). The probe was designed by the coding sequence of 35S and *AtP3B* gene (5′-CCGGAAAC-CTCCTCGGATTC-3′, 5′-ATCTCCAGCGCAAGCTTGTT-3′). Autoradiography was used to detect the hybridization signals.

### Gene expression analysis

Genomic DNA was extracted from putative WT (cv. Xushu 29) and transgenic plant using purified genomic DNA in premix (Bioneer, Korea). The specific primer set based on the sequence of part of the 35S promoter and *AtP3B* gene (5′-CTACAAATGCCATCATTGCG-3′, 5′-CTCTTCCTCCTT-TGTGGCTG-3′) was used in PCR analysis. Total RNA was isolated from the third leaf, of sweetpotato shoot tips, using TRIzol reagent (Invitrogen, USA), and reverse transcribed using TOPscript™ RT DryMIX (dT18) (Enzynomics, Korea) according to the manufacturer’s instructions. The gene-specific primers used for PCR analysis were as follows: the AtP3B primer set (5′-GATGATTGAGCCTGCGATTC-3′, 5′-TTACCCCTTTTCACCAGCAC-3′) was used to amplify a cDNA encoding AtP3B. The total synthesized cDNA was also used to amplify the ubiquitin extension gene (*UBI*) as a reference gene using *UBI* gene-specific primers (5′-TCGACAATGTGAAGGCAAAG-3′, 5′-CTTGATCTTCTTCGGCTTGG-3′) [55]. All quantitative RT-PCR analysis was conducted with Ever-Green 20 Fluorescent Dye (BioFACT, Korea) in a CFX96 Touch Real-time PCR Detection System (MJ Research, USA). All reactions were repeated at least three times.

### Stress treatment of whole plants

WT and OP plants were grown at 25 °C (60% relative humidity, 16 h/8 h [light/dark] photoperiod) in a growth chamber for 1 month and utilized for the temperature stress tolerance assay. For the heat stress experiments, the sweetpotato plants were transferred to a growth chamber maintained at 45 °C for 12 h and then returned to 25 °C for 24 h recovery. For low temperature stress experiments, the plants were transferred to a growth chamber maintained at 4 °C for 48 h and then returned to 25 °C for 24 h recovery.

### Low temperature storage of sweetpotato tuberous roots

WT and OP sweetpotato tuberous roots were harvested, followed by curing. The storage temperature was then changed to 13 °C (optimal storage condition) at relative humidity >80%. Roots were sampled for the control time point (0 week) and at specified internals during an 8 week exposure to storage at optimal storage condition (13 °C) and low temperature storage condition (4 °C), respectively.

### Analysis of photosynthetic activity

The photosynthetic activity was estimated based on the maximal yield of the photochemical reaction in PSII (Fv/fm). The Fv/fm values in the 3rd-4th mature leaves from top of 1-month-old WT and OP plants were measured using a portable Chl Fluorescence Meter (Handy PEA, England) after 30 min of dark adaption

### Analysis of lipid peroxidation

Malondialdehyde (MDA) content, a marker of lipid peroxidation, was determined according to a modified thiobarbituric acid (TBA) method [56]. The MDA content was determined spectrophotometrically at A_532_ and A_600_. The experiments were repeated three times.

### Ion leakage measurements

Ion leakage was measured in root tissues according to Lieberman et al. with minor modifications [57]. The leakage from tissue slices was determined using conductivity measurements of solutions surrounding 40 discs per replication, which were approximately 2 mm thick and 1 cm in diameter. The discs were washed ten times in deionized water and drained. The samples were placed in test tubes containing 20 ml of deionized water at 25 °C for 1 h at a shaking speed of 60 rpm. Conductivity was measured using an ion conductivity meter (S230 SevenCompact™, Mettler Toledo, Switzerland). The values were compared with the total conductivity of the solution after autoclaving at 121 °C for 15 min. The experiments were repeated three times.

### Quantification of hydrogen peroxide (H_2_O_2_)

Hydrogen peroxide (H_2_O_2_) levels were determined according to Velikova et al. with minor modifications [58]. Leaf tissues (100 mg) were ground in liquid nitrogen and extracted with 1 ml of 0.1% (*w*/*v*) TCA. The homogenate was centrifuged at 12,000 rpm for 15 min, and 0.5 ml of the supernatant was combined with 0.5 ml 1 M KI. After a 1 h reaction in darkness, the absorbency of the supernatant was read at 390 nm. The H_2_O_2_ content was calculated using a standard curve. The experiments were repeated three times.

### Antioxidant enzyme activity analysis

The third leaves of sweetpotato plants were homogenized in cold condition with 0.1 M potassium phosphate buffer (pH = 7). The homogenate was centrifuged at 12,000 g for 15 min at 4 °C. The supernatant was used immediately for enzyme assays. POD activity was assayed as described by Kwak et al. [59] using pyrogallol as a substrate. CAT activity was assayed as described in Aebi et al. [60]. The experiments were repeated three times.

### Field cultivation of transgenic sweetpotato plants

Sweetpotato field cultivation was conducted in 2015 at the Living Modified Organism (LMO) field of National Institute of Crop Science, Muan, South Korea. Stems were subsequently cut to 15 cm in length, and 40 stems per line were transplanted in the field with three replications. The field cultivation method was performed according to Park et al. [61]. At harvest time, shoot lengths and the fresh weights of the aerial parts and tuberous roots were recorded.

## Additional file

Additional file 1: Figure S1. Transcript levels of *IbHSP*, *IbCBF,* and *IbCOR* genes in WT and OP plants. qRT-PCR analysis of *IbHSP*, *IbCBF,* and *IbCOR* genes including *IbHSP17.6*, *IbHSP18.2, IbCBF3* and *IbCOR27* was performed using mRNA isolated from sweetpotato leaves. Under normal growth conditions (25 °C), WT and OP plant samples were taken, and the transcript levels of these genes were normalized to tubulin gene (*TUB*) expression. The error bars represent the mean ± SD of three biological replicates. Asterisks indicate significant differences between WT and OP plants by ANOVA at * *p* < 0.05 and ** *p* < 0.01. **Table S1**. Primers used for PCR analysis. (DOCX 473 kb)

## Abbreviations

APX: Ascorbate peroxidase
CaMV: Cauliflower mosaic virus
CAT: Catalase
CBF: C-repeat-binding factor
COR: Cold-responsive
HSP: Heat-shock protein
MDA: Malondialdehyde
MV: Methyl viologen
POD: Peroxidase
qRT-PCR: Quantitative reverse-transcription PCR
ROS: Reactive oxygen species
WT: Wild type
