# Supplementary material for: Overexpression of Arabidopsis P3B increases heat and low temperature stress tolerance in transgenic sweetpotato
Source: BMC Plant Biol. 2017 Aug 14;17:139. doi: 10.1186/s12870-017-1087-2 (PMC5557506; doi:10.1186/s12870-017-1087-2)
Supplement: Additional file 1: Figure S1. — Transcript levels of IbHSP, IbCBF, and IbCOR genes in WT and OP plants. qRT-PCR analysis of IbHSP, IbCBF, and IbCOR genes including IbHSP17.6, IbHSP18.2, IbCBF3 and IbCOR27 was performed using mRNA isolated from sweetpotato leaves. Under normal growth conditions (25 °C), WT and OP plant samples were taken, and the transcript levels of these genes were normalized to tubulin gene (TUB) expression. The error bars represent the mean ± SD of three biological replicates. Asterisks indicate significant differences between WT and OP plants by ANOVA at * p < 0.05 and ** p < 0.01. Table S1. Primers used for PCR analysis. (DOCX 473 kb) [file 12870_2017_1087_MOESM1_ESM.docx]

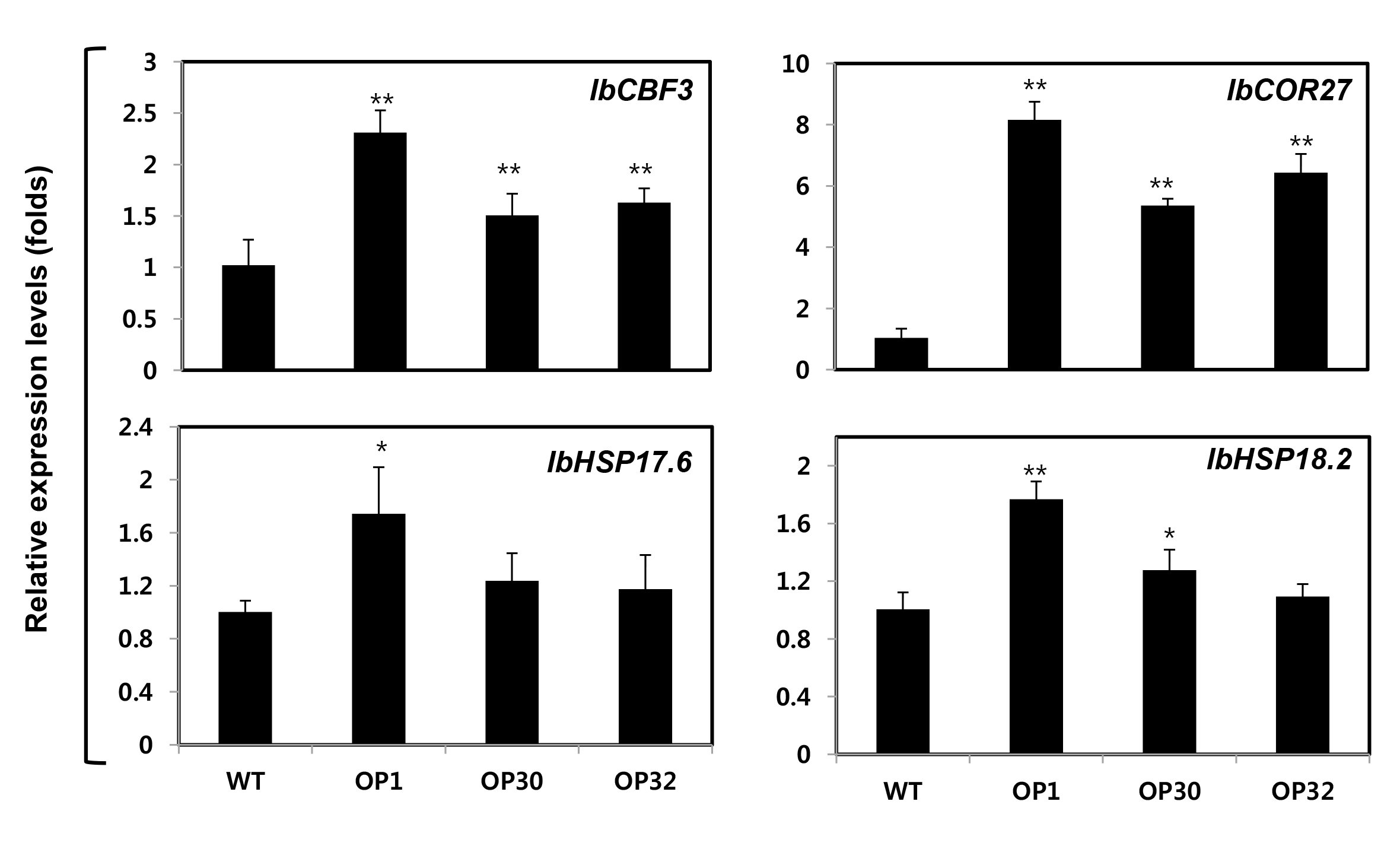


**Figure S1.** Transcript levels of *IbHSP*, *IbCBF,* and *IbCOR* genes in WT and OP plants. qRT-PCR analysis of *IbHSP*, *IbCBF,* and *IbCOR* genes including *IbHSP17.6*, *IbHSP18.2, IbCBF3* and *IbCOR27* was performed using mRNA isolated from sweetpotato leaves. Under normal growth conditions (25°C), WT and OP plant samples were taken, and the transcript levels of these genes were normalized to tubulin gene (*TUB*) expression. The error bars represent the mean ± SD of three biological replicates. Asterisks indicate significant differences between WT and OP plants by ANOVA at * p < 0.05 and ** p < 0.01.

**Table S1. Primers used for PCR analysis.**

| Primer | Sequence (5'-3') | Application |
| --- | --- | --- |
| *IbCBF3-F* | TTCGCCACTGTCTTCTTC | Real-time PCR |
| *IbCBF3-R* | CATATCCTGGACTTCTTGTTG | Real-time PCR |
| *IbCOR27-F* | CCAATCTCAGCTTCCTTTGC | Real-time PCR |
| *IbCOR27-R* | CATGAACCTCAGCATTGTCG | Real-time PCR |
| *IbHSP17.6-F* | CACCACATCATGGACTACGC | Real-time PCR |
| *IbHSP17.6-R* | TCGATCTGCACCTTGATGTC | Real-time PCR |
| *IbHSP18.2-F* | GAGCGGAGCAAAGAGAAAGA | Real-time PCR |
| *IbHSP18.2-R* | TCAATGGCCTTAACCTCTGG | Real-time PCR |
| *TUB-F* | CAACTACCAGCCACCAACTGT | Real-time PCR |
| *TUB-R* | CAAGATCCTCACGAGCTTCAC | Real-time PCR |
